# A case report of successful management of fulminant *Clostridium difficile* colitis post-ileostomy reversal with administration of vancomycin through a transverse colostomy

**DOI:** 10.1186/s40792-019-0744-0

**Published:** 2019-11-21

**Authors:** Keiji Matsuda, Yojiro Hashiguchi, Mitsuo Tsukamoto, Kohei Ohno, Yuka Okada, Takahiro Yagi, Yoshihisa Fukushima, Atsushi Horiuchi, Ryu Shimada, Tsuyoshi Ozawa, Tamuro Hayama, Takeshi Tsuchiya, Junko Tamura, Hisae Iinuma, Keijiro Nozawa, Yuko Sasajima, Fukuo Kondo

**Affiliations:** 1 Department of Surgery, Teikyo University School of Medicine, 2-11-1 Kaga, Itabashi-ku, Tokyo, Japan; 2 Department of Pathology, Teikyo University School of Medicine, 2-11-1 Kaga, Itabashi-ku, Tokyo, Japan

**Keywords:** *Clostridium difficile*, Enteritis, Postoperative, Stoma reversal

## Background

*Clostridium difficile* (CD), discovered in 1935, is one of the most common hospital-acquired infection diseases. Lessa et al. reported that there were 27,300 deaths of 61,400 patients with reported hospital-acquired CD infections (CDI) [1]. Rates of postoperative CDI were reported to be ranging from 0.54 to 2.37% [2]. Skancke et al. reported that stoma reversal was a risk factor for CDI [3]. To the best of our knowledge, there have been no previous reports of treatment with the administration of vancomycin through a transverse colostomy created after CDI.

We report the first case of fulminant type CD colitis post-stoma reversal surgery that underwent transverse colostomy and has survived after intensive care.

## Case presentation

A 58-year-old man underwent laparoscopic-assisted intersphincter resection (ISR) and ileostomy for lower rectal cancer. The pathological finding was pT2, pN0, cM0, pStage I according to the Japanese stage classification of colorectal cancer, the 9th edition [4]. He has no comorbidity. For the convenience of the patient, reversal of ileostomy was planned 1 year after the operation for rectal cancer. Functional end-to-end anastomosis was performed in the ileostomy reversal operation in 111 min without any intraoperative complications.

**Fig. 1:**
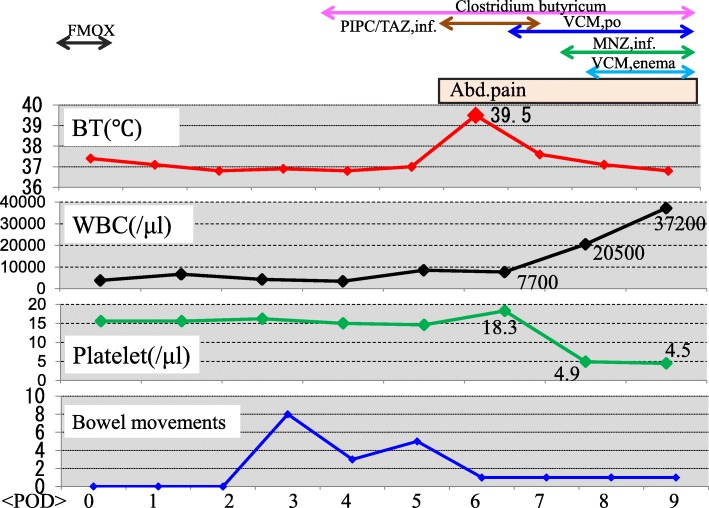
Clinical Course after ileostomy closure

**Fig. 2:**
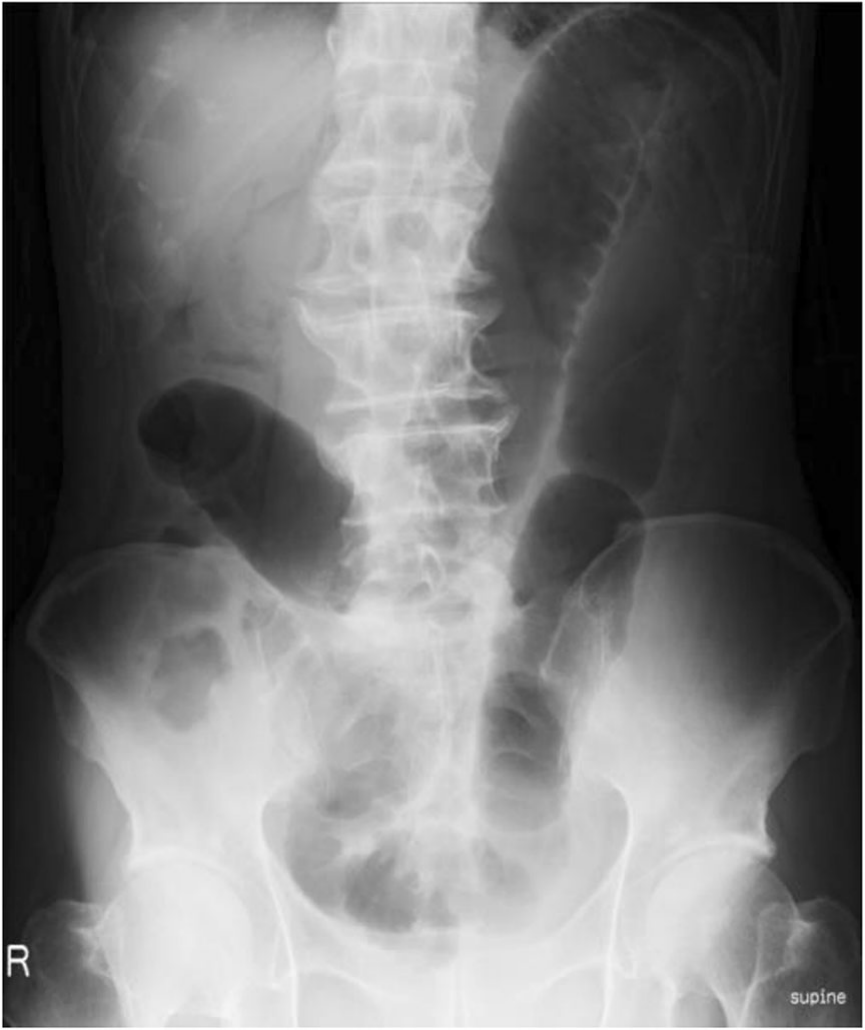
In X-ray on the 6th postoperative day, a gas was seen throughout the dilated colon

**Fig. 3:**
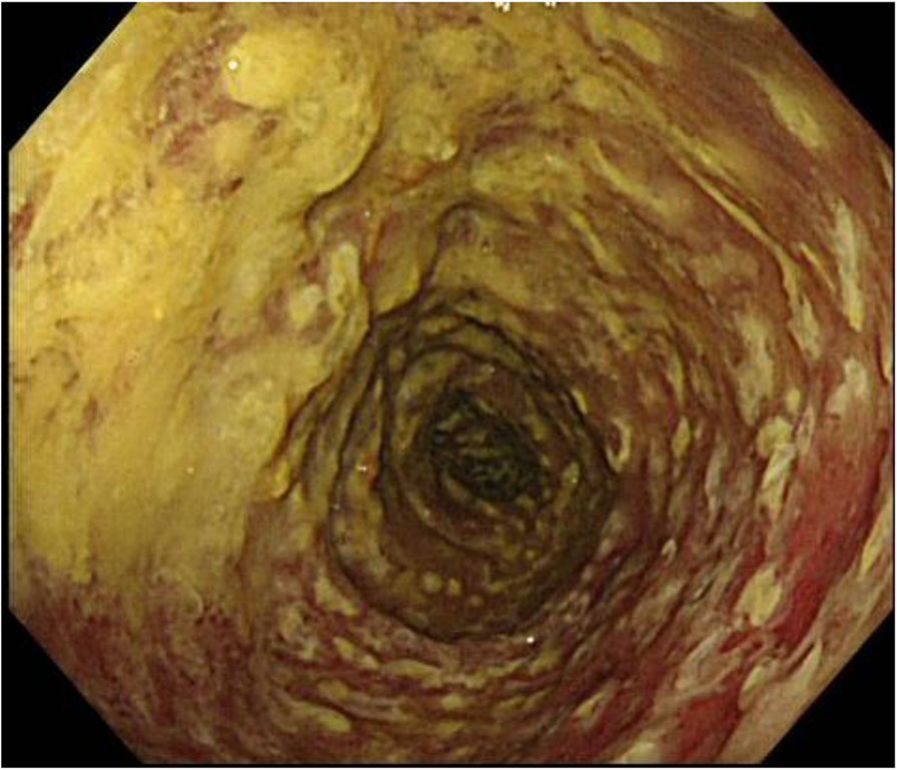
Colonoscopy on the 7th postoperative day revealed the adhesion of white leaves to the whole large intestine

**Fig. 4:**
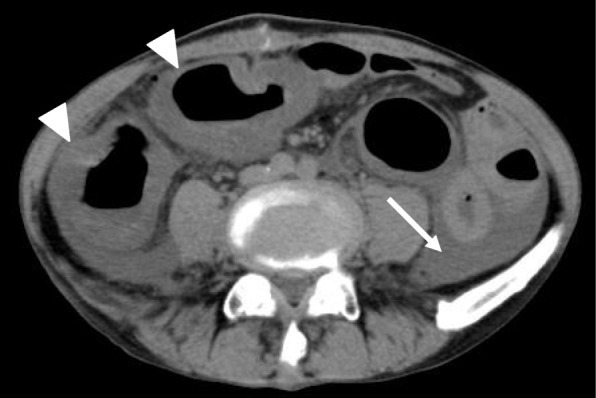
In CT on the 7th postoperative day, ascites (white arrow), colon dilation and colonic wall thickening (white triangle) was evident

**Fig. 5:**
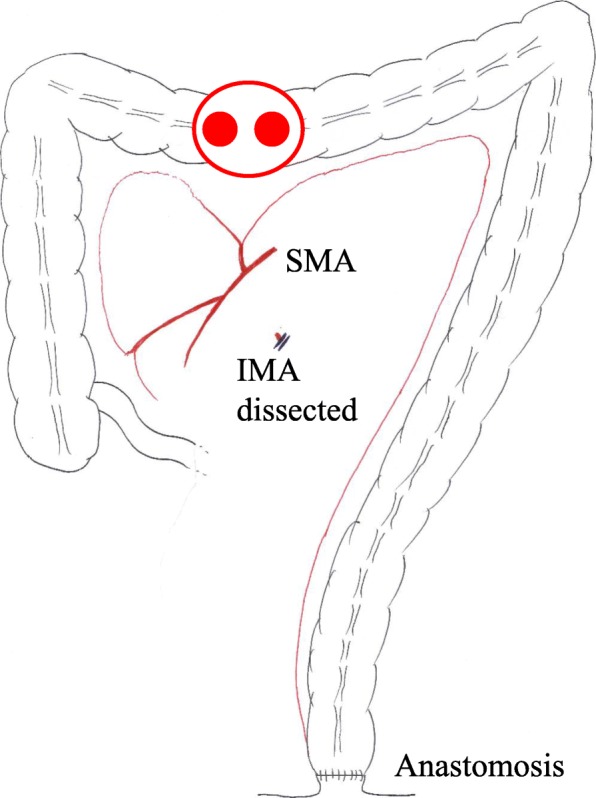
Emergency surgery of transverse colostomy creation was performed

**Fig. 6:**
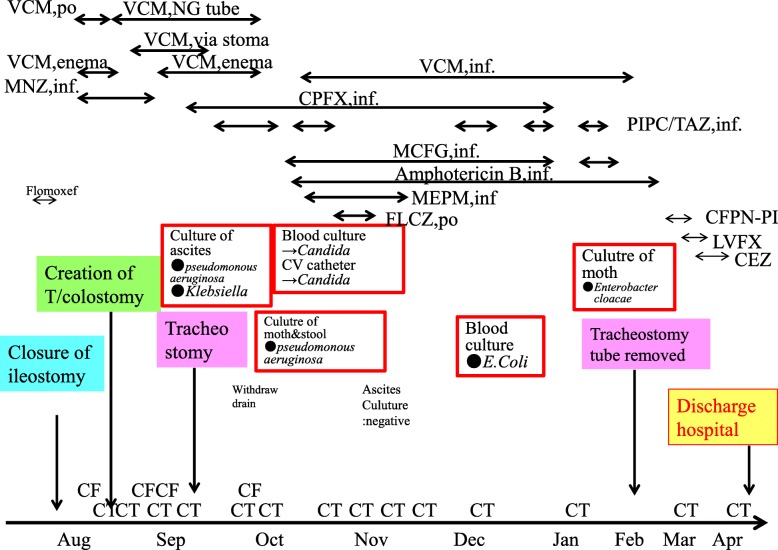
Total clinical course of this patient

Preventative antibiotic “flomoxef sodium” was administered during the operation and on the first postoperative morning. Postoperative course was uneventful until the fifth postoperative day. On the sixth postoperative day, he suffered from abdominal pain and a fever of 39.5 °C (Fig. 1). The defecation was once. White blood cell count was 8500/μl, but CRP was 4.6 mg/dl. In X-ray, gas was seen throughout the dilated colon (Fig. 2). Blood culture test resulted in negative. Antibiotic tazobactam/piperacillin hydrate was started, and nothing per os was ordered. Although abdominal pain did not change on the seventh day, creatinine was inclined to 2.6 mg/dl and CRP to 25.1 mg/dl. Colonoscopy revealed the adhesion of white leaves to the whole large intestine, and the patient was diagnosed with pseudomembranous enteritis (Fig. 3). CD toxin was negative, and glutamate dehydrogenase was positive. Nucleic acid amplification test (NAAT) was not performed because it was not covered by insurance at that time. The culture of stool showed *Klebsiella*, *Proteus*, *Enterococcus*, *Staphylococcus*, and *Corynebacterium*. Vancomycin by oral administration was started. In CT, ascites, colon dilation, and colonic wall thickening was evident (Fig. 4). On the eighth day, white blood cell count rapidly increased to 20,500/μl, and platelets decreased to 49,000/μl (Fig. 1). DIC treatment was simultaneously performed. Colonoscopy examination showed no improvement of pseudomembranous enteritis, and vancomycin enema and metronidazole infusion were added to his medical treatment. CT showed increased ascites on the ninth day. White blood cell count increased to 37,200/μl, platelets decreased to 45,000/μl, and creatinine increased to 5.9 mg/dl. He was diagnosed with fulminant type CD colitis which did not respond to drug treatment. Emergency surgery of transverse colostomy creation was performed for two reasons: one was the aim to administer vancomycin throughout the colon easily, and the other was in too bad a condition for total colectomy (Figs. 5 and 6).

**Fig. 7:**
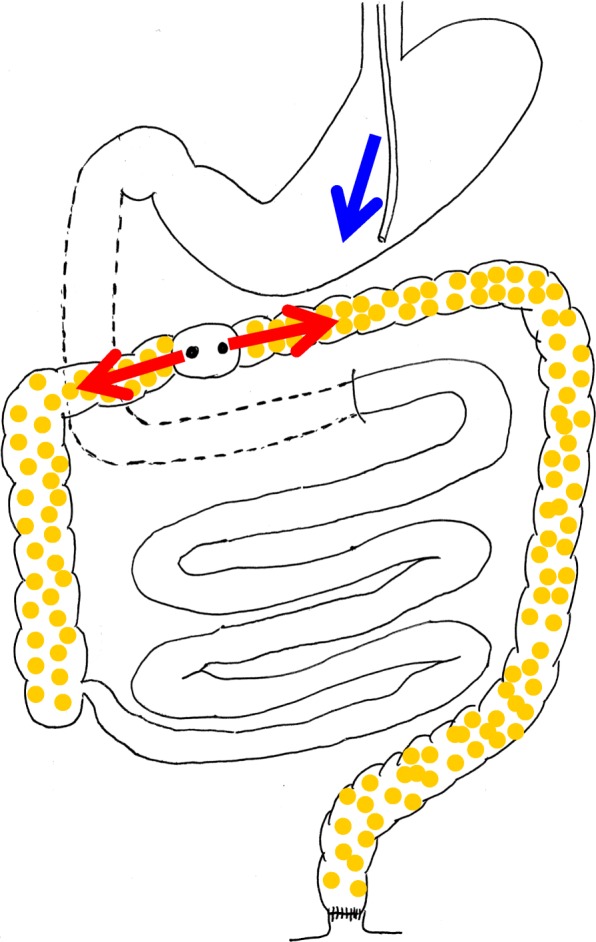
Administration through the nasogastric tube (blue arrow) and the transverse colostomy (red arrows) of vancomycin was performed. Vancomycin was administered to the oral and anal side from the stoma

**Fig. 8:**
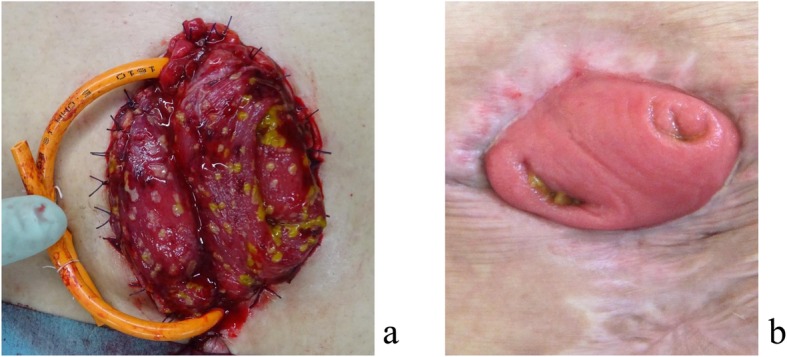
The pseudomembranous change of the transverse colostomy observed just after creation of the stoma (Fig. 8a) disappeared after intensive therapy (Fig. 8b)

During the operation, dilated and edematous transverse colon was observed, and ascites of 2700 cc was aspirated. After the operation, administration through the nasogastric tube and the stoma of vancomycin with infusion of metronidazole was performed (Fig. 7). Vancomycin was administered to the oral and anal side from the transverse colostomy. On the 16th day after the surgery of stoma creation, colonoscopy showed disappearance of the white moss on the oral side of the stoma, but it remained on the anal side. Vancomycin enema from the anus was performed simultaneously. Colonoscopy on the 28th day showed improvement on the anal side of the stoma. Vancomycin administration was terminated on the 40th day. On the 56th day, disappearance of the white moss was observed on the anal side of the anus. White blood cell count normalized on the 10th day and platelet normalized on the 19th day. But pleural effusion, atelectasis, and abundant ascites were seen on the 14th day. Puncture of ascites showed green color, and *Pseudomonas aeruginosa* and *Klebsiella* were detected in culture. In addition, *Candida* was detected and treatment for mycoses was also done. Finally, pleural effusion and ascites disappeared and the pseudomembranous change of the transverse colostomy was cured (Fig. 8). He was discharged 8 months after ileostomy reversal. One year and 3 months have passed after leaving the hospital, and he is doing well at outpatient.

## Discussion

CD, an anaerobic, spore-forming, gram-positive, rod-shaped bacterium, is the most common nosocomial pathogen causing pseudomembranous colitis [5]. Health care-associated CDI and community-associated one were both increasing [6]. The morbidity associated with CDI process is significant, with more than 9% of hospital admissions for CDI resulting in death [6].

According to the guideline [7], factors of CD intestinal infection are diarrhea, fecal CD toxin positive, isolation of toxin-producing CD, and showing findings of pseudomembranous enterocolitis. Sometimes, ileus or toxic megacolon is observed instead of diarrhea, as seen in our case. The definition of severe status stated by each academic society is that at least one of the following factors is observed: temperature > 38.5 °C, white blood cells count > 15,000/μl, increased creatinine, or decreased albumin. In our case, all factors were observed. According to the IDSA guideline, ileus or megacolon is a factor of fulminant type, and both were observed in our case [8].

The clinical symptoms range from mild diarrhea to complicated systemic deterioration known as fulminant colitis (FC). FC is the most feared presentation of CDI and occurs in 2 to 3% of patients. FC accounts for most of the serious CDI complications, such as ileus, megacolon, colonic perforation, and death. Contributory factors associated with FC include age, immune status, patient comorbidities, and microbial virulence factors. It should be noted that diarrhea is minimum in FC patients with ileus, which leads to toxic megacolon [6]. For the treatment of fulminant CD enteritis, vancomycin orally, vancomycin enema, and metronidazole infusion are recommended. All treatments were employed in our case.

According to the algorithm [6], once CDI is suspected, the responsible antibiotic should be discontinued and treatment with oral vancomycin or metronidazole promptly started. Generally, the effect of medical treatment is evaluated every day after its initiation; if it is considered to be ineffective, the indication for surgical treatment should be assessed. For patients requiring an operation, the mean time from onset of symptoms to diagnosis is 5.6 days [9]. The generally accepted current algorithm for CDI reserves the surgical treatment for those with worsening clinical symptoms, deterioration, worsening white blood cells (WBC), cardiopulmonary compromise, or end-organ failure [6]. However, it is apparent that the surgical outcome would be disastrous if the surgery was postponed until the deterioration of the general condition of the patient who was already sick.

In our case, CDI occurred after ileostomy reversal and transverse colostomy was useful. The following sources were searched for studies reporting cases of reported CDI following reversal of ileostomy and undergoing transverse colostomy: Ovid, Embase, and Medline using PubMed. The search terms included (*C.difficile*) AND (reversal of ileostomy), (*C.difficile*) AND (closure of ileostomy), (*C.difficile*) AND (ileostomy) AND (reversal), (*C.difficile*) AND (ileostomy) AND (closure), (*C.difficile*) AND (colostomy) in all fields.

Case reports of CDI occurring after reversal of ileostomy were summarized in Table 1 [9–13]. Our case is thought to be the first case of fulminant CDI occurring after ileostomy reversal that underwent creation of colostomy and was given vancomycin through it. From these results, we judged that our case has rarity and was worth to be reported.

**Table 1:** CDI reported cases after reversal of ileostomy

Author/year	Ref. no.	Age/sex	Background	Duration between onset and surgery (days)	WBC (per μl)	Surgical procedure	Antibiotics after surgery	Outcome
Shen/2009	[10]	61/F	Ulcerative colitis, after total proctocolectomy with IAA and a loop ileostomy	10	61,000	None	VCM po, MNZ inf	Dead, the postoperative 35th day
Nair/2010	[11]	72/M	After LAR with ileostomy	14	17,500	None	VCM po	Alive, 9 months
Abe/2012	[9]	69/M	After LAR with ileostomy	4	9460	Total colectomy with ileostomy	VCM po	Dead, the postoperative second day
Almerie/2015	[12]	60/F	After LAR with ileostomy	4	11,400	None	MNZ po	Alive, 2 weeks
Fashandi/2016	[13]	77/M	After diverting loop ileostomy	3	4500	Total colectomy	Not recorded	Dead, the postoperative fifth day
Our case/2019		58/M	After ISR with ileostomy	6	37,200	Transverse colostomy	VCM NG tube, VCM enema through T/colostomy, MNZ inf	Alive, 15 months

*LAR* low anterior resection, *ISR* intersphincter resection, *MNZ* metronidazole

The incidence of CDI after reversal of ileostomy has been reported. A systematic review of CDI following reversal of ileostomy showed the overall incidence of CDI was 1.8% (242/13728) [14]. Schanke et al. showed the incidence of CDI was 3.04% in stoma reversal and higher than 1.25% in elective colectomy with *p* < 0.001. Randall et al. reviewed retrospectively the incidence of CDI and stated 4.2% in ileostomy reversal, 2.1% in right hemicolectomy, and 1% in anterior resection [15]. Wilson et al. analyzed patients undergoing reversal of loop ileostomy using the Nationwide Inpatient Sample and showed their incidence of CDI was 1.6% (217/13,245) [16]. Usually, ileostomy reversal is not considered as a major surgery, but attention should be paid to ileostomy reversal as a high-risk group for CDI [17].

There is no clear explanation yet for encountering CDI after reversal of ileostomy. CD could colonize the small bowels, with many studies reporting enteritis with CD [18]. Animal studies have shown that excluded colons undergo mucosal and muscular atrophy with derangement in the intestinal immune system [19]. Another potential cause might be due to the reduced nutrition in excluded colons. This could change the unique microbial ecosystem in the large bowel in favor of the more fastidious bacteria such as CD causing colonization of the colon [16]. When the stoma is closed, the spores could get reactivated and enter a growth phase leading to clinical infection.

Many guidelines recommend subtotal colectomy and end ileostomy with preservation of rectum as an operative treatment for CDI [6]. But for fulminant CDI patients, subtotal colectomy would be a heavy burden. As another operative treatment, guidelines recommend diverting loop ileostomy and colonic lavage followed by intravenous metronidazole and vancomycin administered via the efferent limb of the ileostomy [6]. However, vancomycin via the ileostomy might not reach left-sided colon for the long distance. In our case, vancomycin was administered through the transverse colostomy and it was effective. Actually, in our case, subtotal colectomy was thought to be very difficult for two reasons. One was that the patient was too exhausted to undergo a major surgery. The other was that our case had undergone ISR with the anastomosis at the anal canal and the inferior mesenteric artery was divided at the root. Like our case, when the patient who had undergone surgery for rectal cancer with ileostomy suffered from fulminant CDI occurring after ileostomy reversal, creation of transverse colostomy should be considered as one of the treatment options.

## Conclusions

We presented a case with fulminant CD colitis followed by a successful surgical treatment of transverse colostomy for vancomycin administration. It is important to recognize that ileostomy reversal is not only a simple surgery but also a high-risk group of CD colitis. Transverse colostomy could be an alternative for subtotal colectomy and end ileostomy with preservation of the rectum, especially for a patient with physical declines or one after rectal surgery.

## Data Availability

The authors declare that all the data in this article are available within the article.

## Abbreviations

CEZ: Cefazolin
CFPN-PI: Cefcapene pivoxil
*E. coli*: *Escherichia coli*
FLCZ: Fluconazole
FMOX: Flomoxef
IMA: Inferior mesenteric artery
LVFX: Levofloxacin
MCFG: Micafungin sodium
MNZ: Metronidazole
PIPC/TAZ: Piperacillin/tazobactam
SMA: Superior mesenteric artery
VCM: Vancomycin
